# Curcumin and Vitamin D Reduce HMGB-1 mRNA Levels in Mice Infected with Salmonella typhi

**DOI:** 10.21315/mjms2024.31.5.10

**Published:** 2024-10-08

**Authors:** Ami Febriza, Hasta Handayani Idrus

**Affiliations:** 1 Department of Physiology, Faculty of Medicine and Health Sciences, Universitas Muhammadiyah Makassar, Indonesia; 2 Centre for Biomedical Research, Research Organization for Health, National Research and Innovation Agency (BRIN), Cibinong Science Centre, West Java, Indonesia

**Keywords:** curcumin, antimicrobial agent, Salmonella typhi, high-mobility group box-1, vitamin D

## Abstract

**Background:**

This study examined the effects of curcumin and vitamin D on high-mobility group box-1 (*HMGB-1*) mRNA expression in mice infected with *Salmonella typhi*.

**Methods:**

The experimental design allocated 40 mice, intraperitoneally infected with *S. typhi*, to pre- and post-test controls randomly divided into four groups (10 mice per group). Mice in group A were treated with the antibiotic levofloxacin (1.95 mg/kg once daily) as the positive control; group B mice were administered curcumin at a dose of 200 mg/kg body weight; group C mice were treated with a curcumin dose of 200 mg/kg BW and vitamin D; and group D mice received distilled water (placebo) as the negative control. The intervention was performed for 5 days. On day 10, *HMGB-1* mRNA expression was measured, and the results were compared to those before the intervention.

**Results:**

*HMGB-1* mRNA level in group C decreased significantly by 5.76-fold (95% confidence interval: 2.55, 8.98). In contrast, *HMGB-1* mRNA levels did not decrease significantly in group B.

**Conclusion:**

These results suggest that the combination of curcumin and vitamin D reduced *HMGB-1* mRNA levels in infected mice, highlighting the potential of this combination as an antimicrobial and anti-inflammatory agent.

## Introduction

Infectious diseases are major global health concerns. Typhoid fever remains prevalent in low- and middle-income countries because of limited access to clean water, poverty and unhygienic practices (1, 2). Without effective treatment, typhoid fever can have a fatality rate of 10%–30%. In 2000, 21.6 million new cases of typhoid fever, resulting in 210,000 deaths worldwide, were reported (2). Multidrug resistance (MDR) in the management of typhoid fever has emerged as an increasing concern, largely because of the inappropriate use of antibiotics and incorrect disease diagnosis. Consequently, it is becoming increasingly challenging to treat this condition effectively, posing a significant threat to public health (3, 4). Given the high mortality rate, higher number of relapses and MDR cases, many attempts have been made to combine antimicrobial agents with other herbal or active substances to treat *Salmonella* infections (5, 6).

Curcumin exhibits antimicrobial and anti-inflammatory effects. Previous studies have reported that curcumin activates the vitamin D receptor pathway, leading to the expression of cathelicidin antimicrobial peptide (CAMP) mRNA (7, 8). A study has reported that a combination of curcumin and vitamin D inhibits the growth of *S. typhi* in mice (9). In contrast, combining curcumin with cotrimoxazole reduced the efficacy of the antibiotic in both intestinal and extraintestinal organs (10).

During the early stages of a bacterial infection, the immune system recognises the pathogens and releases high-mobility group box-1 (*HMGB-1*), which is crucial for immunity. *HMGB-1* is a damage-associated molecular pattern (DAMP) that activates the innate immune system. Immune cells can secrete *HMGB-1* or dead cells can passively release it. This protein controls inflammation, particularly in response to microbial infections. It mediates the Toll-like receptor (TLR)-4 signalling pathway, which is essential for infectious and non-infectious diseases (11–13). Previous studies have indicated that extracellular *HMGB-1* triggers a harmful immune response, resulting in inflammation. *HMGB-1* contributes to progression of infectious and inflammatory disorders (14, 15). Patients with severe sepsis and experimental animals with sepsis-induced organ dysfunction have elevated *HMGB-1* levels in their system. *HMGB-1* inhibition prevents multiple organ failure and increases survival rates in patients with severe sepsis (16, 17). The levels of *HMGB-1* were significantly elevated in rheumatoid arthritis patients during the active phase and were also associated with asthma severity (18, 19).

A previous study has shown that using an anti-*HMGB-1* antibody is critical for protecting mice from the lethal effects of lipopolysaccharide (LPS)-induced endotoxemia (13, 20). Reducing extracellular and circulating *HMGB-1* levels could be a potential mechanism for preventing sepsis or progression of the inflammatory response during infection. We found that administration of curcumin and vitamin D reduced *HMGB-1* mRNA levels in infected mice, indicating that this combination may be an effective antimicrobial and anti-inflammatory strategy. We aimed to expand on the results of previous studies and examine the effects of curcumin and vitamin D on *HMGB-1* mRNA expression in mice infected with *S. typhi*.

## Methods

### Experimental Animals

In this experiment, we used 40 BALB/c mice aged 9 weeks–10 weeks and weighing 30 g–35 g. All mice were male, healthy and without disabilities. All mice were provided by the Laboratory Faculty of Medicine Universitas Muslim Indonesia, Makassar, Indonesia. The mice were housed in cages of adequate size at 28 **°**C with 40%–60% humidity. The mice had *ad libitum* access to food and water during the experiments. Mice were fed with pellets diets consisting of seeds, cereals, sugars, minerals, vegetable oil and herbs. All mice were intraperitoneally injected with *S. typhi* (3 mL, 10^3^ CFU/mL) (9). The mice were randomly assigned to four groups containing 10 mice each using an online random team generator. Mice in group A were treated with levofloxacin (1.95 mg/kg once daily) as the positive control. Group B mice were administered a curcumin dose of 200 mg/kg of body weight (BW). Mice in group C were treated with a curcumin dose of 200 mg/kg BW and vitamin D (details in the following section). Group D mice received distilled water (placebo) as the negative control. All interventions were administered through the nasogastric sonde once daily for 5 days.

### Curcumin and Vitamin D Preparation

Curcumin was purchased from Merck (curcumin for synthesis; chemical formula [4-(OH)-3-(CH_3_O)C_6_H_3_CH=CHCO]_2_CH_2_, also known as 1,7-bis(4-hydroxy-3-methoxyphenyl)-1.6-heptadiene-3,5-dione, turmeric yellow or diferuloylmethane. The experiment involved dissolving 200 mg/kg of curcumin in a normal saline solution. A curcumin dose of 200 mg/kg BW was employed in a prior study (9). Vitamin D was purchased from Merck (cholecalciferol). The dose of vitamin D was in accordance with the FDA-recommended dose for adults. The dose administered to mice was calculated by applying a conversion factor of 0.0026. Group C received a dose of 0.52 IU of vitamin D and curcumin (200 mg/kg BW) daily for 5 days.

### HMGB-1 mRNA Expression

Real-time quantitative reverse transcription (qRT)-polymerase chain reaction (PCR) using SYBR Green qRT-PCR Supermix (Bio-Rad, Hercules, CA, USA) was performed to determine *HMGB-1* mRNA expression. An RT-PCR thermocycler (Namnay CFX connect; CFX96 Touch Real-Time PCR; Bio-Rad Laboratories, Hercules, CA, USA) was used to measure mRNA expression (21). Nucleic acid was extracted according to a previous study (22). One hundred microlitre of blood samples were obtained from all groups and evaluated for mRNA expression at baseline and on days 4 and 10. The forward and reverse primers used for *HMGB-1* were GAAGTTGGACCCCAATGC and TCATCTGCTGCAGTGTTGTTCC, respectively, with Gene ID 15289 (23). mRNA expression was assessed using *GAPDH* as the reference. The forward and reverse primers for *GAPDH* were GACCACAGTCCATGCCATCA and CATCACGCCACAGTTCC, respectively.

In the present study, 2.5 μL of DNA extract was introduced to a mixture of primary PCR mix and primers, constituting a total volume of 22.5 μL. The first stage of amplification was carried out at a temperature of 94 °C for 2 s, followed by 40 cycles of amplification, each consisting of 60 s at 94 °C and 45 s at 57 °C. The expression levels of the *HMBG-1* gene were calculated by determining the difference between the threshold cycle number (Ct) of the GAPDH gene and the Ct of *HMBG-1*, which was then raised to the power of two. It is important to note that Ct values are defined as the number of PCR cycles at which the fluorescent signal during the PCR reaches a fixed threshold.

### Statistical Analyses

The data were analysed using SPSS version 26.0 software (IBM Corporation, Armonk, NY, USA) and tested using the Shapiro-Wilk test. One-way ANOVA was performed to compare the numerical mean differences among the groups. Paired *t*-tests were used to compare mean *HMGB-1* mRNA expression levels in the groups before and after the intervention. *P* < 0.05 was considered statistically significant.

## Results

Three days after infecting all the mice intraperitoneally with *S. typhi*, mice in group A were administered levofloxacin, those in group B with curcumin, those in group C with curcumin and vitamin D, and those in group D with a placebo. There were no significant differences in age, weight and *HMGB-1* mRNA expression among the four groups at baseline (Table 1).

Paired-sample *t*-test was performed to determine statistically significant mean differences in *HMGB-1* expression before and after the intervention. Following the intervention, *HMGB-1* mRNA expression reduced in all groups. *HMGB-1* mRNA level in group C decreased significantly by 5.76-fold (95% confidence interval: 2.55, 8.98). Conversely, mean differences were not statistically significant, indicating no significant decrease in *HMGB-1* mRNA levels, in group B (Table 2).

**Figure 1:**
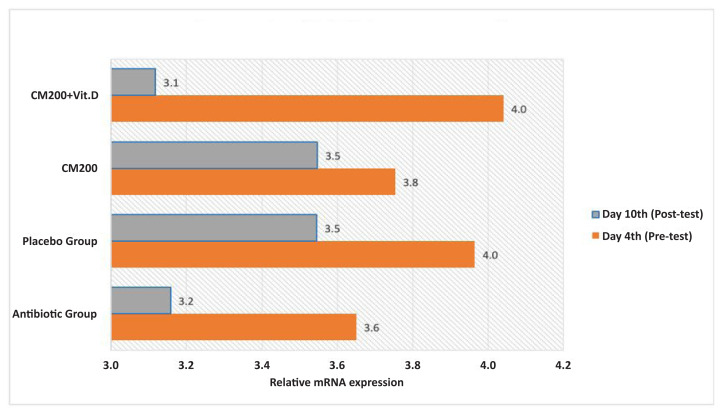
Changes in *HMGB-1* mRNA expression at the time of observation, before intervention day 4 (pre-test) and after the intervention day 10 (post-test). CM200: group receiving curcumin (200 mg/kg body weight); CM200+Vit D (200 mg/kg body weight curcumin and 0.52 IU Vitamin D); placebo group (distilled water); antibiotic group (levofloxacin 1.95 mg/kg body weight)

*HMGB-1* mRNA expression decreased in all groups after the intervention (Figure 1), with some noticeable variations. *HMGB-1* mRNA expression increased in all groups after infection with *S. typhi* (day 4). Group C showed the most significant decrease in *HMGB-1* mRNA levels, which was almost similar to the levels observed in group A.

## Discussion

Typhoid fever is a bacterial infection caused by *S. typhi* that primarily affects the intestine. The infection can spread through contaminated food or water, leading to severe symptoms, such as pyrexia, abdominal pain and diarrhoea (24). When *S. typhi* enters a host, it is destroyed by macrophages. *S. typhi* LPS induces macrophage signalling (25, 26), leading to inflammation. *HMGB-1* is a late-phase mediator of inflammation triggered by proinflammatory cytokines. It induces immunosuppressive and pathological effects following cytokine release during infection (26). To the best of our knowledge, this study is the first in vivo intervention to explore the potential benefits of curcumin and vitamin D in reducing *HMGB-1* mRNA expression in *S. typhi*-infected mice.

*HMGB-1* levels in the context of infection have been previously evaluated. Animal studies using infected mice have reported high *HMGB-1* levels after infection. Mice intraperitoneally infected with *Klebsiella pneumoniae* showed a significant increase in average *HMGB-1* mRNA expression (9.64-fold change; *P* < 0.05) (27). Animal models of systemic endotoxemia have identified *HMGB-1* as a late mediator of sepsis, with mice showing increased serum *HMGB-1* levels 8 h–32 h after endotoxin exposure (28). Furthermore, clinical studies have reported that *HMGB-1* levels are higher in patients with infection, particularly with pneumonia, peritonitis or severe sepsis (29, 30). Another study revealed *HMGB-1* levels between patients with asthma and healthy subjects; the levels were significantly higher in patients with asthma (31). To reduce mortality caused by bacterial infection, it is essential to modulate the immune response by decreasing *HMGB-1* secretion. A previous study found that nuclear *HMGB1* triggers the immune response to fight Classical Swine Fever Virus (CSFV) replication. CSFV’s N^pro^ protein avoids this antiviral immunity by increasing *HMGB-1*’s acetylation, which moves it from the nucleus to the cytoplasm and then to the lysosome for degradation (32).

In general, theoretical and experimental evidence supports the notion that curcumin inhibits microbial growth. It has antibacterial effects against both Gram-negative and Gram-positive bacteria (9, 33, 34). Moreover, it is a potential alternative to antibiotics for the treatment of typhoid fever. Our findings demonstrated that the combined administration of curcumin at a dose of 200 mg/kg and vitamin D resulted in a significant decrease in *HMGB-1* mRNA expression in *S. typhi*-infected mice (Table 2). Curcumin binds to vitamin D receptors, leading to the production of the antimicrobial peptide cathelicidin (7, 8), which contributes to innate immunity. Cathelicidin damages the bacterial membrane and neutralises LPS production by bacteria by decreasing bacterial viability (35, 36). Previous studies have reported that antimicrobial peptides can inhibit the binding of LPS to target cells, thereby suppressing the release of *HMGB-1* and necrotic cell death (37). In addition, a significant decrease was observed in group A (Table 2). Antibiotic-induced bacterial reduction decreases LPS levels and downregulates *HMGB-1* expression (38).

## Conclusion

There was a significant mean difference in *HMGB-1* mRNA expression in infected mice treated with the combination of curcumin and vitamin D. Mice that received curcumin and vitamin D exhibited lower *HMGB-1* mRNA expression levels than those in the placebo group. It is important to note that this study has some limitations. It focuses on only a limited number of markers and has been conducted exclusively in animal settings. Further research is required to determine the optimal dose of a combination of curcumin and vitamin D that can be administered to humans.

## Figures and Tables

**Table 1:** Characteristics of experimental animals

Variables	Groups				*P*-value*

	Group A (antibiotic)	Group B (CM200)	Group C (CM200 + vit. D)	Group D (Placebo)	
Mice age (in weeks)	9.30 ± 1.06	9.70 ± 1.25	9.10 ± 1.10	9.10 ± 0.99	0.585
Mice body weight (g)	33.34 ± 3.04	33.98 ± 3.04	34.23 ± 3.16	34.36 ± 1.66	0.854
*HMGB-1* mRNA expression (fold-change) at baseline	6.31 ± 0.54	5.94 ± 0.52	6.25 ± 0.40	6.00 ± 0.53	0.285

Notes:

* Mean comparison between groups using one-way ANOVA;

Data was reported as mean ± SD; CM = curcumin

**Table 2:** Differences in mean level of *HMGB-1* expression (fold-change) among groups during the intervention

Groups	*HMGB-1* expression (fold-change)		*P*-value*

	Day 4 (pre-test)	Day 10 (post-test)	
Group A (antibiotic)	23.08 ± 0.80	19.97 ± 1.61	0.001
Group B (CM200)	22.33 ± 4.83	21.11 ± 0.58	0.431
Group C (CM200 + vit. D)	25.28 ± 3.16	19.51 ± 1.55	0.003
Group D (placebo)	23.87 ± 2.09	21.35 ± 2.45	0.065

Notes:

* Mean comparison between groups using paired *t*-test;

Data was reported as mean ± SD; CM: = curcumin
